# Anesthetic management for cesarean section in a patient with uncorrected double-outlet right ventricle

**DOI:** 10.1186/s40064-016-2075-y

**Published:** 2016-04-06

**Authors:** Juan Gu, Yunxia Cai, Bin Liu, Sheng Lv

**Affiliations:** Department of Anesthesiology, West China Second University Hospital, Sichuan University, 20#, Section 3 Renmin Nan Road, Chengdu, 610041 Sichuan China; Department of Anesthesiology, West China Hospital, Sichuan University, 37#, Guo Xue Xiang, Chengdu, 610041 Sichuan China

**Keywords:** Double-outlet right ventricle (DORV), Anesthesia, Cesarean section

## Abstract

**Background:**

We describe the anesthetic management for cesarean section in a pregnant woman with uncorrected double-outlet right ventricle. The anesthetic method, treatment of complications and lessons are discussed.

**Case presentation:**

A 28-year-old woman visited our emergency room for progressive dyspnea and recurrent hemoptysis at 30 weeks’ gestation. Her New York Heart Association functional class was III–IV. Echocardiography indicated that she had congenital heart disease of double-outlet right ventricle. She hadn’t received any treatment. The obstetrician decided to terminate the pregnancy by cesarean section. We chose epidural anesthesia and pumped phenylephrine at the same time to minimize hemodynamic fluctuation. Just in the process of changing position to supine position with uterus displaced to the left, the patient coughed badly and complained about dyspnea. At the same time, the oxygen saturation decreased quickly. The symptoms were ameliorated soon by treating as heart failure. But the symptoms reappeared after oxytocin administration. At the end of surgery, the baby was sent to the Neonatal Intensive Care Unit for premature birth. The mother recovered successfully and discharged 7 days later.

**Discussion and Evaluation:**

Double-outlet right ventricle is a seldom disease and pregnancy with uncorrected double-outlet right ventricle is rare. In this case, the patient belonged to the type of ventricular septal defect characterized by subaortic ventricular septal defect without pulmonary stenosis. Most of the aorta arises from the right ventricle, the volume of venous blood was injected from the right ventricle into the aorta, which decided the oxygen saturations. Compared to general anesthesia, epidural anesthesia reduces venous return and alleviates the cardiac burden. So, we pumped phenylephrine along with epidural anesthesia in case of critical cyanosis following significant blood pressure decrease. In the process of anesthesia, dyspnea, cough and cyanosis attacked the patient for two times. The most probable reason was heart failure which induced by the sudden increasing of returned blood. As a result of severe cough, pulmonary artery pressure increased rapidly. Then a great amount of venous blood was injected into the aorta and cyanosis became more serious as well as oxygen saturation declined quickly.

**Conclusion:**

Epidural anesthesia with continuous phenylephrine infusion is a preferable choice for parturient women with uncorrected double-outlet right ventricle for cesarean section. It is not the optimal choice for this type of patients to lie on the left. The dose of oxytocin should be reduced to avoid potential cardiovascular complications.

## Background

The incidence of pregnancy with cardiac disease ranges from 0.4 to 4.1 %, and congenital heart disease takes up a large proportion (McFaul et al. 1988). Double-outlet right ventricle (DORV) is a rare congenital heart disease. The incidence is approximately 0.5–0.8 per 10,000 births (Pradat et al. 2003). DORV is a congenital cardiac malformation in which both pulmonary artery and aorta predominantly arise from the right ventricle, and ventricular septal defect (VSD) always coexists (Lev et al. 1972). Signs of heart failure often appear when the patient is young, so this disease is often diagnosed and treated early (Lacour-Gayet 2008). Own to the improvement in surgery intervention, women who suffer from congenital heart anomalies are more likely to live to the age of child-bearing (Gianopoulos 1989). On the other hand, the heart is usually too fragile to endure the physiological changes during pregnancy, which will put them at risk. The patient in this report was a pregnant woman with uncorrected DORV who was scheduled for cesarean section. Because of complex pathologic changes, it is challenging for anesthesiologists to manage appropriately.

## Case presentation

**Fig. 1:**
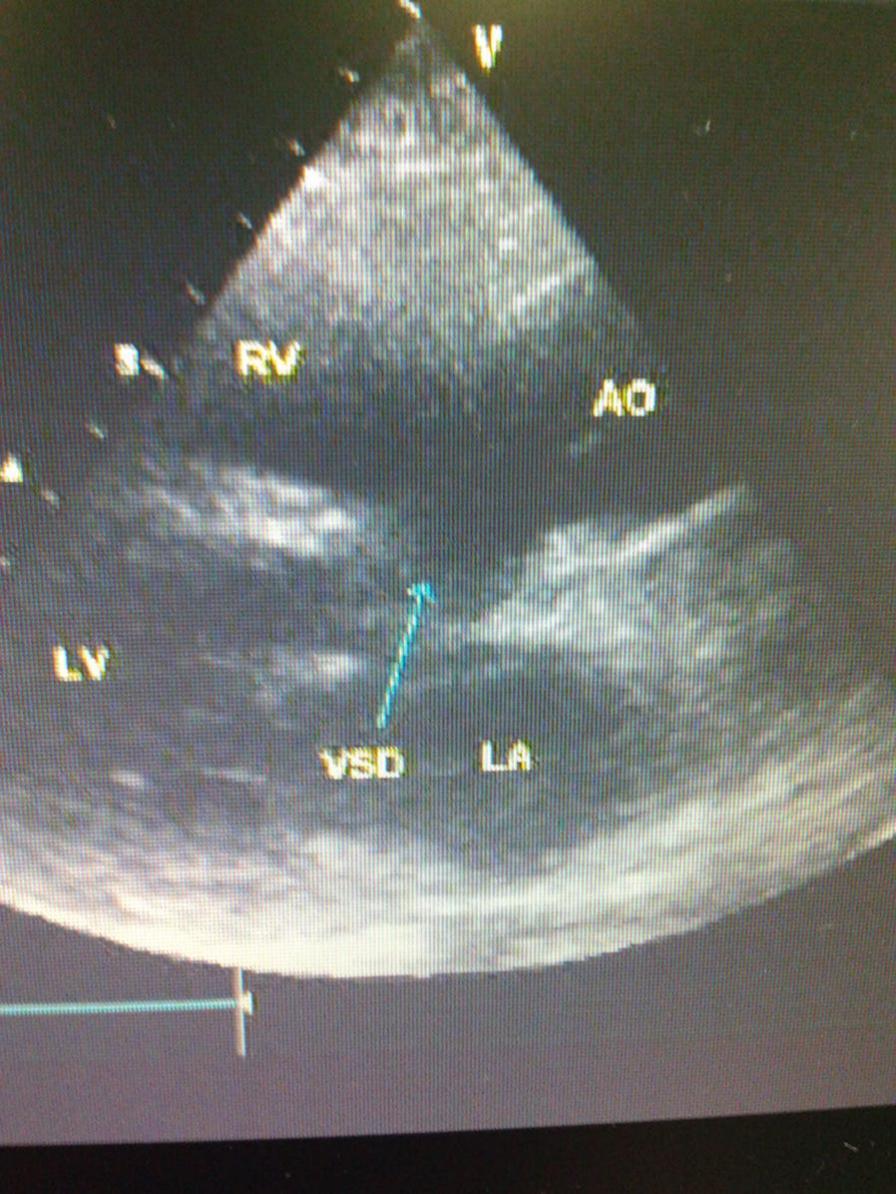
Echocardiogram of the pregnant woman. Cardiac function: EF was 58 %, FS was 30 %. Dimensional and M echocardiograph: enlargement of right atria and ventricular (RA 55 cm, RV 30 cm); the aorta and pulmonary artery were parallel without enlargement; the aorta ridded on ventricular septum (80 % was from right ventricular); the defect of ventricular septum was 26 mm which was under aorta; there was absence of fibrous joint between anterior mitral valve leaflet and the posterior wall of aorta. Color Doppler ultrasound: there was a left to right shunt between ventricularis was detected (V_max_ was 1.6 m/s)

A 28-year-old pregnant woman (height 155 cm; weight 55 kg) visited our emergency room complaining about progressive dyspnea and recurrent hemoptysis at 30 weeks’ gestation. Two years ago, she was diagnosed to have congenital heart disease because of heart failure which occurred at her fist pregnancy. As a result, she had to have an abortion at 20 weeks’ gestation. But she refused to receive any treatment. She said that she was asymptomatic except for mild cyanosis before getting pregnant. This was her second pregnancy. She said she was uncomfortable on the left lateral position during late pregnancy this time. Physical examination indicated cyanosis, orthopnea and acropachy. Temperature was 36.6 centigrade, blood pressure was 115/60 mmHg, heart rate was 99 per minute, and pulse oxygen saturation (SpO_2_) was 75 % when she inhaled air. Electrocardiogram showed incomplete right bundle branch block. Chest radiograph revealed bilateral patch shadows, cardiac enlargement and bulging of pulmonary artery segment. Echocardiography of the mother showed: The diameter of the right atria and right ventricular were 55 cm and 30 cm separately; the diameter of the left atria and left ventricular were normal; the aorta and pulmonary artery were parallel without enlargement; the aorta ridded on ventricular septum, 80 % of the aorta was from right ventricular; there was a 26 mm ventricular septum defect under aorta; there was a left to right shunt between left and right ventricles was detected, the max velocity was 1.6 m/s; there was absence of fibrous joint between anterior mitral valve leaflet and posterior wall of aorta; the ejection fraction of the left ventricle was 58 %. The diagnosis of the echocardiography was DORV (subaortic ventricular septal defect),mild reflux of pulmonary valve and contraction function of left ventricle was normal (Fig. 1). Echocardiography of the embryo indicated cor triatriatum of right atrium. The results of laboratory tests were normal except for platelet count which was 79 × 10^9^/L. The hemoglobin concentration was 112 g/L. Her New York Heart Association (NYHA) functional class was III–IV. The patient was treated with oxygen, furosemide and dexamethasone when she arrived at our hospital. The dexamethasone was used to promote maturity of the feta’s lung. One day’s later, the symptoms of the patient were improved. The obstetrician decided to terminate pregnancy by cesarean section for deterioration of the heart function.

When she arrived at the operational room, oxygen 8 L/min was provided with a mask. The operating table was turned to reverse trendelenburg position. She was monitored with pulse oximetry, 5-lead ECG and invasive blood pressure Measures (SPACELAMBS, UltraviewSl2700). One 16-G access was placed in the peripheral venous. We should had inserted the central venous catheter. But unfortunately, we failed after trying twice. Because the patient couldn’t lie down and the ultrasound machine was unavailable. Ringer’s lactate was administered slowly according to the heart rate and blood pressure. In order to balance pulmonary circulation and systemic circulation and in case of heart failure. We decided to perform epidural anesthesia and pump phenylephrine at the same time. She was kept on the left lateral position with hips and knees flexed to assist puncturing. One epidural catheter was inserted into L3–L4 interspace with pointed toward caudal direction, another epidural catheter was inserted into L1–L2 interspace with pointed toward cephalic direction. Then the patient was placed on supine position with uterus pushed to the left. Just in the process of changing position, the patient coughed badly as well as complained about chest congestion and dyspnea. At the same time, the SpO_2_ decreased to 50 %. Rale was auscultated on bilateral chest, the blood pressure was 102/65 mmHg. The symptom improved soon after 10 mg furosemide was given intravenously and with the head of bed elevated. 2 % lidocaine 3 mL was injected into epidural for testing. Then sensory block level of S5–T6 was achieved by titrating 15 mL chloroprocaine through the two catheters. Along with epidural anesthesia, phenylephrine was pumped at a rate of 0.1–0.3 μg kg^−1^ min^−1^ to keep hemodynamics stable. During cesarean section, the mother was infused intravenously 5 IU dose of oxytocin after the baby was delivered. 3 min later, the symptoms as they occurred in position changing before reappeared unexpectedly. Then 10 mg furosemide, 3 mg morphine and 2 mg cediland were administered, then the patient felt better gradually. Apgar scores of the baby were 7 at 1 min and 10 at 5 min. In the surgery, the patient received 200 mL Ringer’s lactate, blood loss was about 200 mL and output of urine was 700 mL. The mother was transferred to intensive care unit, the baby was sent to the Neonatal Intensive Care Unit for premature birth postoperatively. 7 days later, the patient recovered and was discharged.

## Discussion

DORV has been divided into different types by the Society of Thoracic Surgeons Database. In this case, the patient belonged to the VSD (ventricular septal defect) characterized by subaortic VSD without pulmonary stenosis. Because the VSD is near to the aorta, the most of the oxygenated blood is ejected into the aorta. The oxygen saturation rests upon the amount of deoxygenated blood ejected from the right ventricle into the aorta. As a patient with a large VSD, the pulmonary over circulation will induce pulmonary hypertension and heart failure. DORV of this type is often diagnosed in infancy for cyanosis and symptoms of congestive heart failure. DORV is a seldom disease and pregnancy with uncorrected DORV is rare. There are only few reports describing the anesthetic management of pregnant woman who suffered from DORV (Ito et al. 2002; Andau et al. 2004). Different from the patients in the previous reports, this patient hadn’t received surgery repair and had more serious symptoms. In addition, poor NYHA functional class and cyanosis were risk factors for maternal or neonatal complications (Siu et al. 2001; Pradat et al. 2003). So, proper anesthetic management was important.

The patient in this report didn’t have obvious symptom until at 30 weeks’ pregnancy. In addition, there was no pulmonary hypertension was indicated in the echocardiography. One possible reason was that the pulmonary circulation and systemic circulation of this patient were balanced very well. But following volume increasing in the third trimester of pregnancy, signs of heart failure appeared as it reported in early studies (Szekely et al. 1973). To this patient, progressive dyspnea and recurrent hemoptysis were the symptoms of cardiac decompensation. Compared to general anesthesia, epidural anesthesia reduces venous return and alleviates the cardiac burden. If general anesthesia was chosen, the drugs used for general anesthesia might suppress the heart function which was already fragile. The mother was in the condition of hypoxia which would result in hypoxia of the baby. The drugs used in general anesthesia induction might worsen the hypoxia of the newborn. Hemoptysis occurred in the patient repeatedly, tracheal intubation might induce bucking and hemoptysis. Epidural anesthesia is easier to keep hemodynamic stability than spinal anesthesia. In addition, we were good at do epidural anesthesia for pregnant woman with heart disease. So we decided to perform epidural anesthesia. Catheters were placed both in L3–L4 and L1–L2 for ensuring adequate analgesia. Most of the aorta arises from the right ventricle, the volume of venous blood was injected from the right ventricle into the aorta, which decided the oxygen saturations (Spaeth 2014). So, we pumped phenylephrine along with epidural anesthesia in case of critical cyanosis following significant blood pressure decrease.

In the process of anesthesia, dyspnea, cough and cyanosis attacked the patient for two times. The symptoms were relieved successfully and quickly by diuretic and cardiotonic. The most probable reason was heart failure which induced by the sudden increasing of returned blood. The first episode occurred when the position was changing. Before the process of epidural puncturing, the patient was placed in position of side-lying with hips and knees flexing, the veins of low limbs were compressed and the venous return were reduced. When the legs unbended and, most importantly, with uterus displaced to the left, the returned blood increased suddenly and heart failure occurred. Cough and dyspnea were the symptoms of pulmonary edema. As a result of severe cough, pulmonary artery pressure increased rapidly. Then a great amount of venous blood was injected into the aorta and cyanosis became more serious as well as oxygen saturation declined quickly. This hypothesis is consist with the patient’s complaint about discomfort when she lay on the left lateral in pregnant period. Cardiac output was 5 % higher in the position of tilting ≥15° than in supine position (Lee et al. 2012). The symptoms recurred after oxytocin was given. Because with a uterine contraction, additional 500 mL blood will return to heart (Maitra et al. 2010). For women with cardiac disease, just 0.1 IU doses of oxytocin could induce adverse effects (Langesaeter et al. 2010). The patient in this report should have been given lower dose oxytocin that was both effective and leaded to fewer complications (Carvalho et al. 2004).

## Conclusions

In conclusion, a pregnant woman with uncorrected DORV for cesarean section was successfully managed by epidural anesthesia and continuous phenylephrine infusion. The lessons we learned from this case were that it is not the optimal choice for this type of patients to lie on the left. The dose of oxytocin should be reduced to avoid potential cardiovascular complications. In addition, we should have placed the patient in sitting position when performing intrathecal anesthesia.

## Abbreviations

DORV: double-outlet right ventricle
VSD: ventricular septal defect
SpO_2_: oxygen saturation
NYHA: New York Heart Association
